# Prevalence of Incidental Pancreatic Cysts on 3 Tesla Magnetic Resonance

**DOI:** 10.1371/journal.pone.0121317

**Published:** 2015-03-23

**Authors:** Patricia Bedesco de Oliveira, Andrea Puchnick, Jacob Szejnfeld, Suzan Menasce Goldman

**Affiliations:** Department of Imaging Diagnosis, Escola Paulista de Medicina, Universidade Federal de São Paulo (UNIFESP), São Paulo, SP, Brazil; H. Lee Moffitt Cancer Center & Research Institute, UNITED STATES

## Abstract

**Objectives:**

To ascertain the prevalence of pancreatic cysts detected incidentally on 3-Tesla magnetic resonance imaging (MRI) of the abdomen and correlate this prevalence with patient age and gender; assess the number, location, and size of these lesions, as well as features suspicious for malignancy; and determine the prevalence of incidentally detected dilatation of the main pancreatic duct (MPD).

**Methods:**

Retrospective analysis of 2,678 reports of patients who underwent abdominal MRI between January 2012 and June 2013. Patients with a known history of pancreatic conditions or surgery were excluded, and the remaining 2,583 reports were examined for the presence of pancreatic cysts, which was then correlated with patient age and gender. We also assessed whether cysts were solitary or multiple, as well as their location within the pancreatic parenchyma, size, and features suspicious for malignancy. Finally, we calculated the prevalence of incidental MPD dilatation, defined as MPD diameter ≥ 2.5 mm.

**Results:**

Pancreatic cysts were detected incidentally in 9.3% of patients (239/2,583). The prevalence of pancreatic cysts increased significantly with age (p<0.0001). There were no significant differences in prevalence between men and women (p=0.588). Most cysts were multiple (57.3%), distributed diffusely throughout the pancreas (41.8%), and 5 mm or larger (81.6%). In 12.1% of cases, cysts exhibited features suspicious for malignancy. Overall, 2.7% of subjects exhibited incidental MPD dilatation.

**Conclusions:**

In this sample, the prevalence of pancreatic cysts detected incidentally on 3T MRI of the abdomen was 9.3%. Prevalence increased with age and was not associated with gender. The majority of cysts were multiple, diffusely distributed through the pancreatic parenchyma, and ≥ 5 mm in size; 12.1% were suspicious for malignancy. An estimated 2.7% of subjects had a dilated MPD.

## Introduction

Pancreatic cysts comprise a wide spectrum of conditions, ranging from benign to frankly malignant lesions, including pre-malignant precursors of pancreas adenocarcinoma, such as intraductal papillary mucinous neoplasm and mucinous cystic neoplasm; therefore, an asymptomatic pancreatic cyst may represent a treatable precursor of invasive carcinoma, which justifies the growing interest in pancreatic cystic lesions [1,2].

Pancreatic ductal adenocarcinoma is one of the more lethal malignant neoplasms, since more than 80% of cases develop metastases or unresectable locoregional invasion at the time of diagnosis, with a 5-year survival rate of only 5% for all stages combined [2,3]. Only a small percentage of patients—i.e., those who detected pancreatic cancer in the initial stages, when there is no evidence of local infiltration—may have a long-term survival after surgical resection. Thus, early diagnosis is the best way of improving this dismal prognosis [3,4].

However, due to the lack of diagnostic tumor markers and to the difficult access to pancreas, there are no strategies for the early detection of pancreatic cancer in the general population. Hence, the identification of predictive signs of this disease and a proper patients’ follow-up are crucial [4]. With the growing use of sectional imaging methods and the technological improvement, the number of incidentally detected pancreatic cystic lesions is increasingly higher [2,5–9].

Although the risk of malignancy is higher in symptomatic pancreatic cysts, malignant or pre-malignant lesions, such as intraductal papillary mucinous neoplasm and mucinous cystic neoplasm, may account for up to 47% of asymptomatic pancreatic cysts [6,10–12]. Thus, it is important to detect these entities in their earlier stages and to suggest the most appropriate management, based on imaging characteristics, a task in which radiologists play a pivotal role [13].

Few studies in the literature evaluated the prevalence of incidental pancreatic cysts in sectional imaging examinations. Zhang et al. reported a prevalence of 19.6% of pancreatic cysts on magnetic resonance imaging (MRI), but their study included patients with history of known or suspected pancreatic disease.

In a recent investigation by Laffan et al. [2], the prevalence of incidental pancreatic cysts on the 16-slice contrast-enhanced computed tomography (CT) was 2.6%. Although the improvement in the technology of CT detectors has increased their conspicuousness in the detection of pancreatic cysts, MRI is capable of detecting a greater number of lesions, due to its inherent higher contrast resolution of soft tissues and its particularly high effectiveness in showing structures containing fluids; thus, it is considered the best imaging modality for the detection of these lesions [1,14]. In addition, MRI cholangiopancreatography allows for achieving a more detailed evaluation of the lesion with regard to how it affects the pancreatic ductal system [14]. In turn, the prevalence of pancreatic cysts in patients undergoing autopsy ranges from 24.3% to nearly 50% [12,13].

Finally, knowing the prevalence of pancreatic cystic lesions in patients with no suspected or known history of pancreatic disease is important for understanding the natural history of these lesions and for guiding its appropriate management [1].

Pancreatic cysts can be identified and characterized using 1.5T MRI; however, the identification provided by routine abdominal protocols is inferior to that obtained with 3T scanners, which have higher spatial and temporal resolution, thus enabling reduced slice thickness and increasing the ability to detect small cysts. Thus, the aim of the present study was to determine the prevalence of incidental pancreatic cysts on 3-tesla MRI in a large sample of patients.

## Materials and Methods

This was a retrospective analysis of 2,678 reports of abdominal MRI scans performed at the Center of Ultrasound and Applied Radiology (Centro de Ultrassonografia e Radiologia Aplicada, CURA), São Paulo, Brazil, from January 2012 to June 2013. Subsequent MRI reports from the same patient were previously excluded from the study, as well as reports of examinations whose pancreas evaluation was impaired by artifacts.

Ninety-five reports were excluded because they were related to patients with known history or suspected pancreatic disease (acute or chronic pancreatitis or presence of pancreatic mass), history of pancreatic surgery, or hereditary conditions associated with presence of pancreatic cysts, to ensure that the pancreatic cysts found on MRI were incidental. Therefore, the presence of pancreatic cysts was assessed in the remaining 2,583 patients and correlated with gender and age of the patients, who were divided into the following age groups: ≤ 39, 40–49, 50–59, 60–69, 70–79, 80–89, and 90–99 years.

Among the patients who were found to have pancreatic cysts, we calculated the number of patients with one cyst and the number of patients with two or more cysts, as well as the number of cysts < 5 mm and ≥ 5 mm. We also assessed their location within the pancreas and the presence of signs suspicious for malignancy. Location was classified as: head, body, tail, neck, uncinate process, diffuse, or two or more sites. The following signs were considered suspicious for malignancy: parietal/septal thickening, parietal/septal enhancement, mural nodules, and/or restricted diffusion.

We also calculated the percentage of patients with a main pancreatic duct (MPD) diameter ≥ 2.5 mm.

This study was approved by the Research Ethics Committee of Universidade Federal de São Paulo and by CURA. There was no need of presenting a signed written consent form because the patients had already allowed their examinations to be used in scientific research at the time of examination. Patient records/information was anonymized and de-identified prior to analysis.

Examinations were performed using a 3 Tesla scanner with a 43 mT/m gradient (Magnetom Verio; Siemens Medical Systems, Erlangen, Germany). The body radiofrequency coil, present in the equipment itself, was used for excitation, and phase-array coil, combined with column coil, were used for MRI signal reception. There was no need of respiratory monitoring.

Abdominal MRI scans were obtained from unique T2-weighted sequences, half-Fourier turbo spin-echo (HASTE) sequences, and in- and out-of-phase T1 CSI images. Table 1 summarizes the physical parameters of the sequences analyzed in this study. MRI reports were made using a workstation operated by an expert in abdominal radiology with more than 20 years of experience in abdominal MRI and a special interest in the pancreas and its conditions.

**Table 1:** Parameters for the magnetic resonance sequences used in the protocol for 3 Tesla scanner.

Sequence	No. of images	Thickness (mm)	TR (ms)	TE (ms)	Matrix	FOV (mm)
Axial T2 HASTE	25	3.0	2000	83	167 x 256	280–350
Axial T2 HASTE Fat Sat	25	3.0	2000	83	167 x 256	280–350
Coronal T2 HASTE	20	3.0	2000	83	167 x 256	280–350
Sagittal T2 HASTE	20	3.0	2000	83	167 x 256	280–350
In-phase axial T1 CSI	35	3.0	173	2.2	167 x 256	280–350
Out-of-phase axial T1 CSI	35	3.0	173	1.1	167 x 256	280–350
Gd Axial VIBE T1	118	1.8	3.23	1.3	179 x 256	280–350

Abbreviations: Fat Sat = fat saturation; VIBE = volumetric interpolated breath-hold examination); Gd = Gadolinium; TR = repetition time; TE = echo time; FOV = field of view.

Cysts were preferentially identified on T2-weighted sequences, on axial and coronal slices, with and without fat saturation. The most common MRI appearance of cysts was that of a small, regularly contoured, thin-walled, T2-hyperintense lesion.

**Fig 1:**
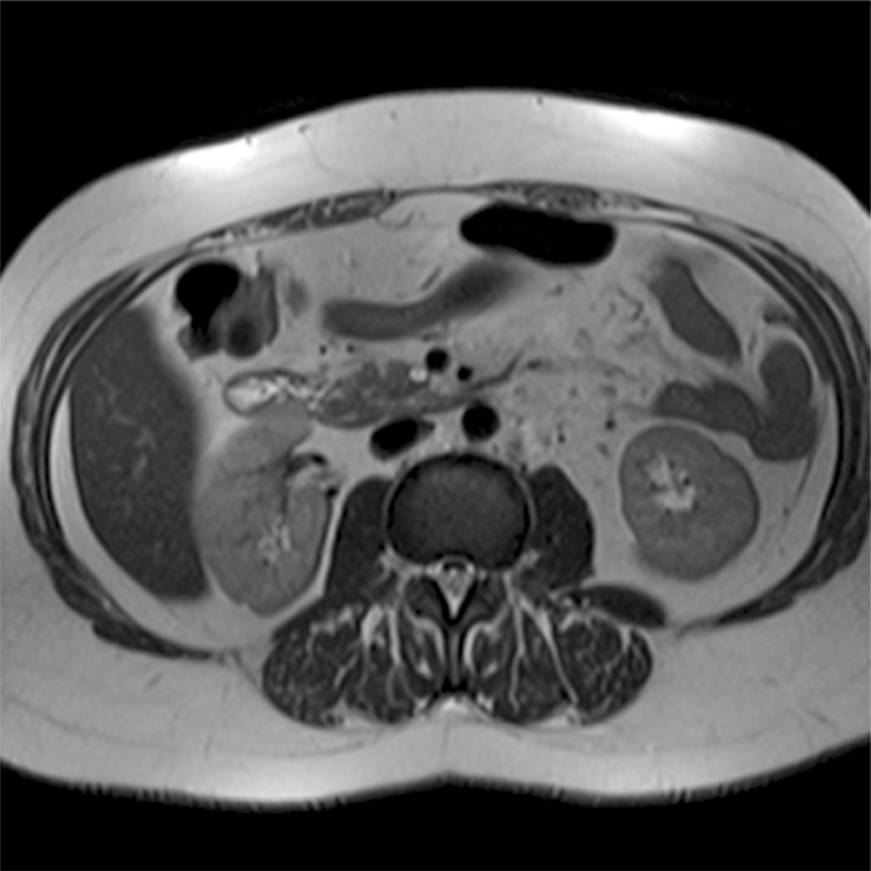
Abdominal MRI (T2-weighted sequence, axial slice), showing a small cyst with thin septum located in the uncinate process of the pancreas.

**Fig 2:**
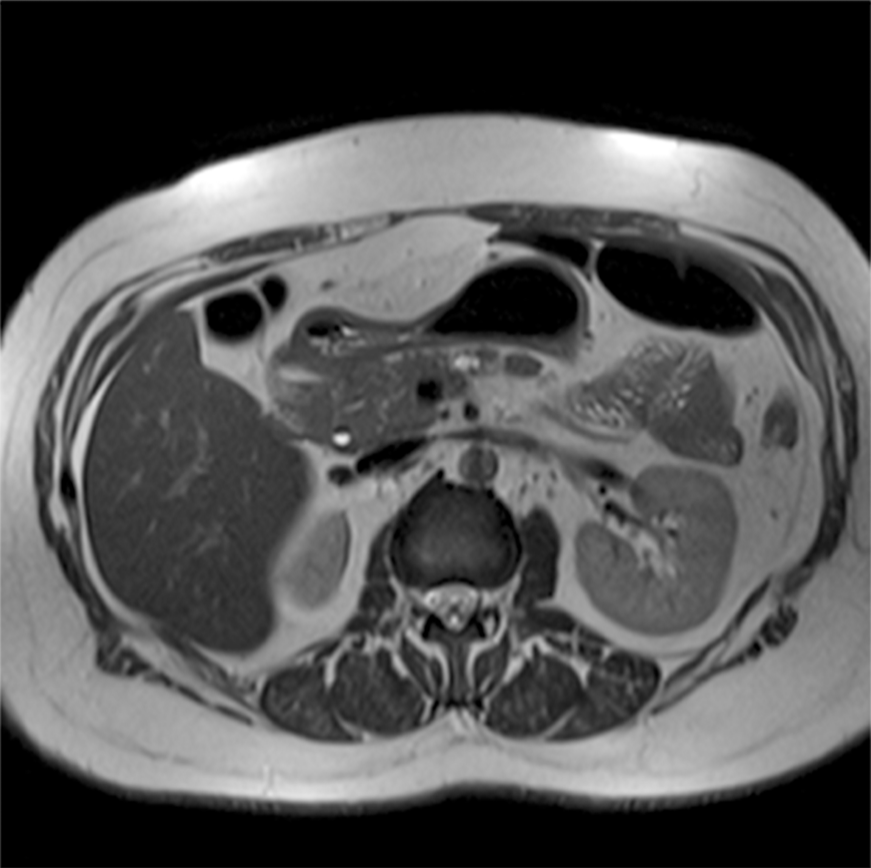
Abdominal MRI (T2-weighted sequence, axial slice), showing a lobulated cyst located in the body of the pancreas.

**Fig 3:**
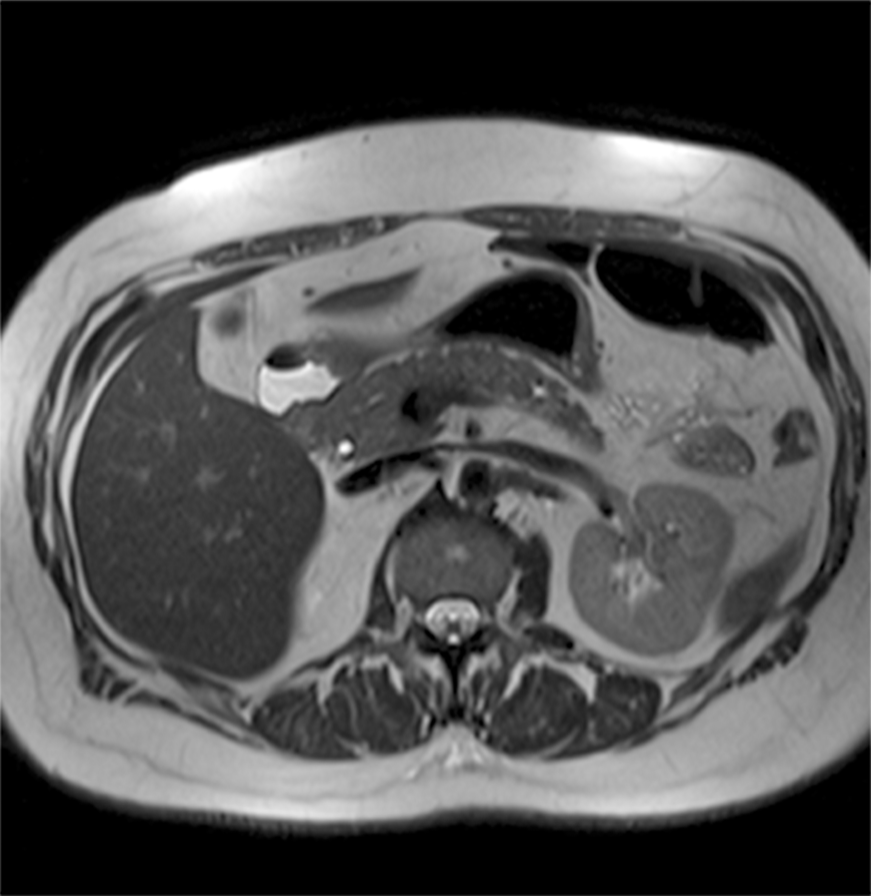
Abdominal MRI (T2-weighted sequence, axial slice), showing an additional small cyst located at the transition between body and tail of pancreas. Same patient as Fig. 2.

**Fig 4:**
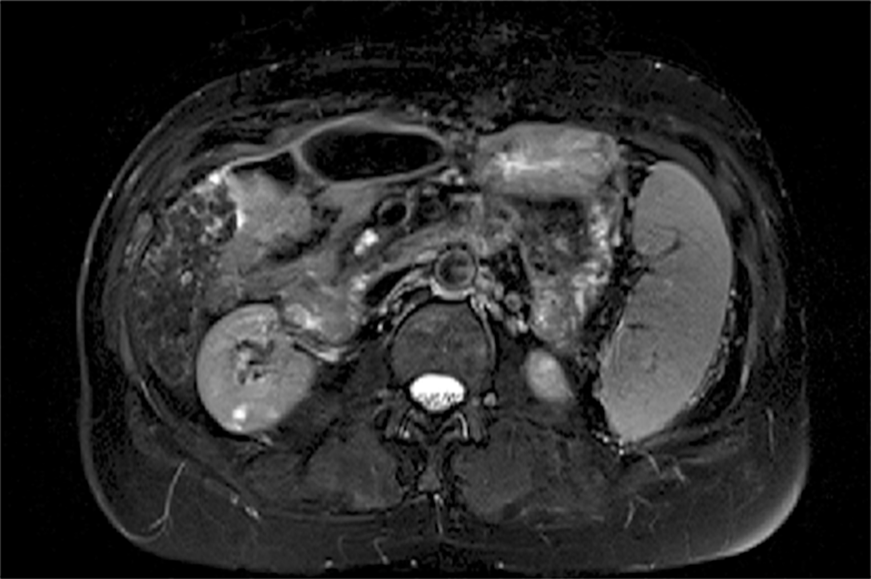
Abdominal MRI (T2-weighted sequence with fat saturation, axial slice), showing a lobulated cyst located in the uncinate process of the pancreas.

**Fig 5:**
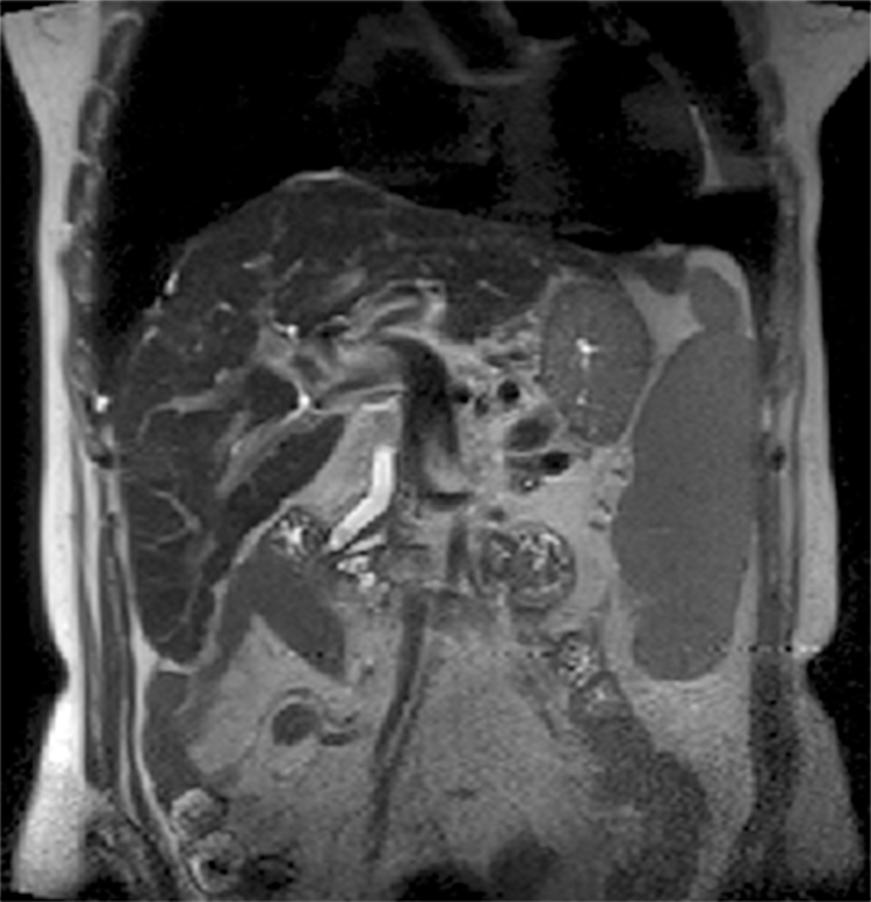
Abdominal MRI (T2-weighted sequence, coronal slice), showing a lobulated cyst within the uncinate process of the pancreas, with slight parietal thickening. Same patient as Fig. 4.

**Fig 6:**
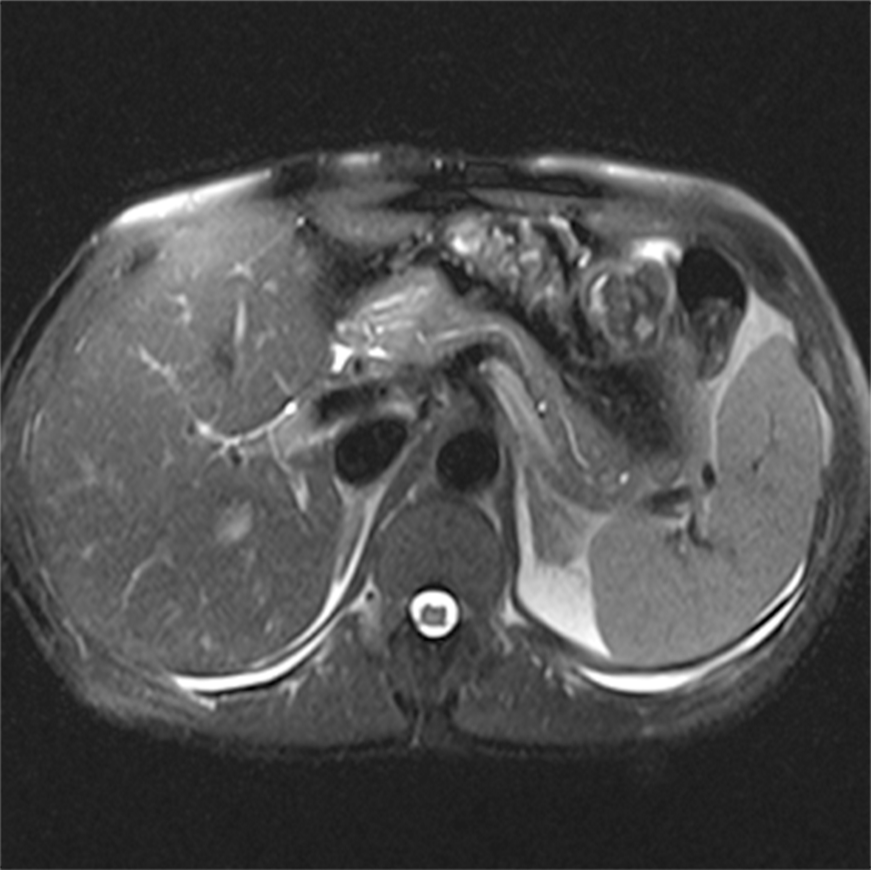
Abdominal MRI (T2-weighted sequence with fat saturation, axial slice), showing two small cysts, one in the tail and one in the transition between body and tail of pancreas.

The following images are representative of pancreatic cysts identified in our sample (Figs. 1–6).

Initially, all variables were analyzed by descriptive analysis. All data were expressed as absolute numbers and percentages (%) or as mean and standard deviation, according to the type of variable. Inferential analysis was performed using the chi-squared test, or the exact Fisher’s test and the Student’s t test for independent variables, with the purpose of confirming or refuting evidence found in the descriptive analysis. The level of significance was set at 5% for all analyses, i.e., results with a p-value lower than 0.05 (*P*<0.05) were considered significant.

Data were entered on Excel 2007 spreadsheets for the proper storage of information. Statistical analyses were performed using the Statistical Package for Social Sciences (SPSS) software (SPSS Inc., Chicago, USA), version 16.0 for Windows.

## Results

Our study population was composed of 2,583 patients, 74.1% of which were women, with ages between 7 and 91 years and mean age of 47.28 ± 15.02 years. Of these patients, 9.3% (239/2,583) showed at least one pancreatic cyst on MRI.

Of the 239 patients with one or more pancreatic cysts, 102 (42.7%) had one cyst and 137 (57.3%) had two or more cysts. Regarding cyst size, 44 patients (18.4%) had cysts < 5 mm and 195 (81.6%) had cysts ≥ 5 mm.

Of these patients with at least one pancreatic cyst, 9 (3.8%) had a cyst located in the head of the pancreas, 44 (18.4%) in the body of the pancreas, 22 (9.2%) in the tail, 10 (4.2%) in the neck, 21 (8.8%) in the uncinate process, 100 (41.8%) had cysts distributed diffusely throughout the pancreas, and 33 had cysts in two or more locations (13.8%).

Furthermore, 29 of the 239 patients with at least one cyst (12.1%) had signs suspicious for malignancy, such as parietal/septal thickening, parietal/septal enhancement, mural nodules, and/or restricted diffusion.

**Fig 7:**
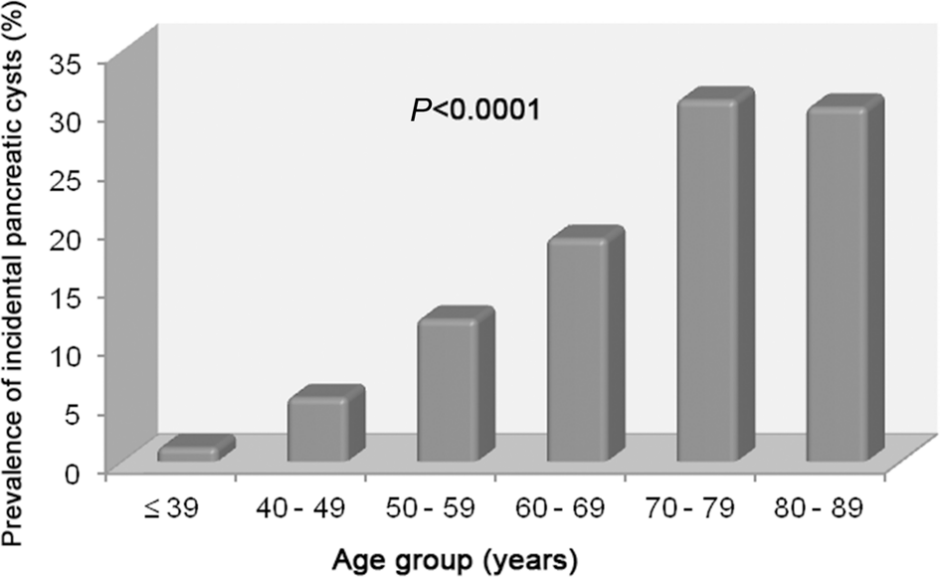
Prevalence of incidental pancreatic cysts according to age.

Mean age of patients with incidental pancreatic cysts was significantly higher (61.13 ± 12.36 years) than that of patients without cysts (45.87 ± 14.54 years) (*P*<0.0001). The prevalence of pancreatic cysts increased significantly with age, affecting 1.2% (11/920), 5.5% (31/568), 12.1% (63/519), 19.0% (69/363), 30.8% (49/159), and 30.2% (16/53) of individuals in the age groups ≤ 39, 40–49, 50–59, 60–69, 70–79, and 80–89 years, respectively (*P*<0.0001, Fig. 7). The age group from 90 to 99 years included only one patient, who did not show any pancreatic cysts.

**Fig 8:**
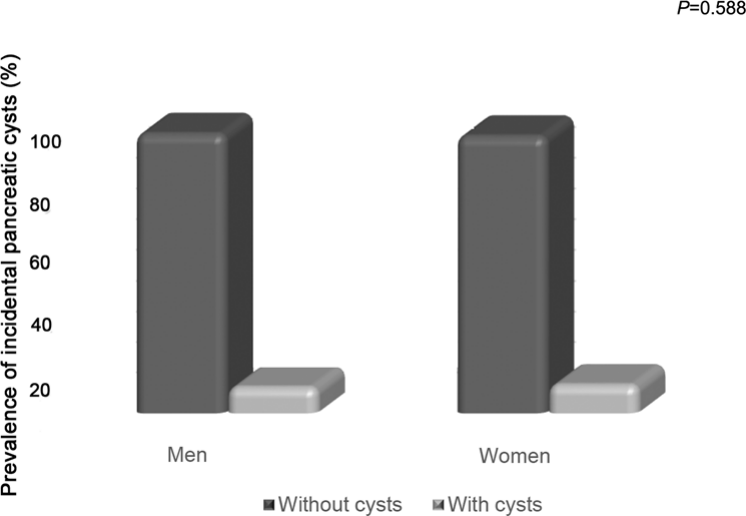
Prevalence of incidental pancreatic cysts according to gender.

There was no significant difference between males and females with regard to the prevalence of pancreatic cysts, which was 8.6% (58/670) among men and 9.4% (181/1913) among women (*P* = 0.588, Fig. 8).

The clinic where the study was conducted includes a center of excellence for women’s health, which explains the female predominance of the sample. Nevertheless, the prevalence of pancreatic cysts was calculated separately for males and females and found to be statistically similar between the two groups, which corroborates previous reports in the literature.

Finally, of the 2,583 patients in the sample, 70 (2.7%) had an MPD diameter ≥ 2.5 mm (dilated), whereas the remaining 2,513 (97.3%) had an MPD diameter < 2.5 mm (not dilated).

## Discussion

The prevalence of pancreatic cysts in the literature ranges from 0.21% in an ultrasound study to 50% in a study with autopsy cases [12, 15]. In our study, the prevalence of incidental pancreatic cysts on MRI was 9.3%.

In the investigation by Zhang et al [9], the prevalence of pancreatic cysts on MRI was 19.6%. However, patients with pancreatic disease or history of pancreas surgery were not excluded from the analysis. If patients with pancreatic masses or history of pancreatitis were excluded from their study, the prevalence of pancreatic cysts would be 13.3%, similar to our results (9.3%).

The prevalence of incidental pancreatic cysts on 16-slice contrast-enhanced CT was 2.6% in the study by Laffan et al. [2]. This difference from our study may be attributed to the increased sensitivity of MRI to detect pancreatic cysts, due to the fact that it provides a higher contrast resolution of soft tissues and structures containing fluids, especially in T2-weighed images [9, 16].

In the study by Lee et al [1], the prevalence of incidental pancreatic cyst on MRI was 13.5%. However, their study was conducted in adult patients, using a 1.5 Tesla MRI scanner. If our study considered only patients aged above 40 years, the prevalence of incidental pancreatic cysts would be 13.7% (228/1,663).

The population of the present study comprised 2,583 patients, with a mean age of 47.28 ± 15.02 years (range, 7–91 years), 74.1% of whom were women. This increased prevalence of female participants is explained by the fact that the study clinic includes a center of excellence for women’s health.

Of the patients included in the study, 9.3% (239/2,583) had at least one pancreatic cyst on MRI, which represents the overall prevalence of incidental pancreatic cysts after excluding patients with a known or suspected history of pancreatic conditions, a history of pancreatic surgery, or hereditary conditions associated with the presence of pancreatic cysts.

Of the 239 patients with one or more pancreatic cysts, 102 (42.7%) had one cyst and 137 (57.3%) had two or more cysts. Regarding cyst size, 44 patients (18.4%) had cysts < 5 mm and 195 (81.6%) had cysts ≥ 5 mm. It is now known that the presence of pancreatic cysts is an independent predictor of pancreatic cancer [4].

Of these patients with at least one pancreatic cyst, 9 (3.8%) had a cyst located in the head of the pancreas, 44 (18.4%) in the body of the pancreas, 22 (9.2%) in the tail, 10 (4.2%) in the neck, 21 (8.8%) in the uncinate process, 100 (41.8%) had cysts distributed diffusely throughout the pancreas, and 33 had cysts in two or more locations (13.8%).

Therefore, most cysts were multiple, 5 mm or larger, and distributed diffusely throughout the pancreas. The body of the pancreas was the second most common cyst location.

Furthermore, 29 of the 239 patients with at least one cyst (12.1%) had signs suspicious for malignancy, such as parietal/septal thickening, parietal/septal enhancement, mural nodules, and/or restricted diffusion.

Of the 2,583 patients that comprised our sample, 70 (2.7%) had a main pancreatic duct (MPD) diameter ≥ 2.5 mm, and 2,513 (97.3%) had an MPD < 2.5 mm (not dilated). MPD dilatation is another independent risk factor for the development of pancreatic cancer [4].

Our study revealed an increase in the prevalence of pancreatic cysts with age, confirming previous results of studies with CT, MRI, and autopsy cases [1, 2, 9, 12]. The prevalence of pancreatic cysts was 1.2% in patients aged ≤ 39 years (11/920), a value significantly lower than the 30.2% found in patients aged between 80 and 89 years (16/53) (*P*<0.0001). Therefore, pancreatic cysts may be an acquired condition that develops with age, which would explain the relative scarcity of cases of pancreatic cysts in the young population.

In agreement with previous reports found in the literature, our results suggest that patient’s gender was not related to the development of incidental pancreatic cysts [1, 9]. The percentage of male and female patients with pancreatic cysts (8.6% vs. 9.4%) was not significantly different (*P* = 0.588). The higher prevalence of females in this study (74.1%) results from the fact that CURA includes in its structure a referral center for women.

Although some studies based on direct visual assessment have shown a higher prevalence of pancreatic cysts compared to CT and MRI reports [1, 17] it is important to point out that our investigation was based on reports from an expert in abdominal radiology with more than 20 years of experience that did not take imaging aspects into account, i.e., even simple and very small cysts were reported.

Our study was limited by its retrospective design, because, since patients underwent MRI due to different clinical indications, the MRI protocol used in this investigation was not specific for pancreas. However, the quality of image obtained from 3 Tesla scanner sequences did not make it difficult to identify cysts larger than 3 mm.

It is widely known that the incidence of pancreatic cancer and cystic diseases of the pancreas is rising in response to an aging population, increasingly widespread use of cross-sectional imaging methods, and advances in imaging technology. Knowledge of the prevalence of these pancreatic abnormalities may contribute to the development of a protocol for rational follow-up of these patients and, thus, to the early diagnosis and prevention of these conditions. In addition, our findings may serve as a reference for future studies.

## Conclusions

This study showed that the prevalence of incidental pancreatic cysts in patients undergoing MRI is 9.3%. This finding increases with age and is not related to patient’s gender.

Most cysts were multiple (57.3%), distributed diffusely throughout the pancreas (41.8%), and 5 mm or larger in size (81.6%). Furthermore, 12.1% of patients with cysts had some feature suspicious for malignancy, such as parietal/septal thickening, parietal/septal enhancement, mural nodules, and/or restricted diffusion.

Overall, 2.7% of patients in the sample had a dilated MPD (diameter ≥ 2.5mm).
